# Myeloid-Derived Suppressor Cells as a Potential Biomarker and Therapeutic Target in COVID-19

**DOI:** 10.3389/fimmu.2021.697405

**Published:** 2021-06-18

**Authors:** Marianna Rowlands, Florencia Segal, Dominik Hartl

**Affiliations:** ^1^ Novartis Institutes for BioMedical Research (NIBR) Translational Medicine, Cambridge, MA, United States; ^2^ Novartis Institutes for BioMedical Research (NIBR), Translational Medicine, Basel, Switzerland; ^3^ Department of Pediatrics I, University of Tübingen, Tübingen, Germany

**Keywords:** COVID-19, immunology, immunity, MDSC, biomarkers

## Abstract

Clinical presentations of COVID-19 are highly variable, yet the precise mechanisms that govern the pathophysiology of different disease courses remain poorly defined. Across the spectrum of disease severity, COVID-19 impairs both innate and adaptive host immune responses by activating innate immune cell recruitment, while resulting in low lymphocyte counts. Recently, several reports have shown that patients with severe COVID-19 exhibit a dysregulated myeloid cell compartment, with increased myeloid-derived suppressor cells (MDSCs) correlating with disease severity. MDSCs, in turn, promote virus survival by suppressing T-cell responses and driving a highly pro-inflammatory state through the secretion of various mediators of immune activation. Here, we summarize the evidence on MDSCs and myeloid cell dysregulation in COVID-19 infection and discuss the potential of MDSCs as biomarkers and therapeutic targets in COVID-19 pneumonia and associated disease.

## Introduction

It has been more than a year since the initial reports of an outbreak of pneumonia in the Hubei province of China, and the subsequent identification of a novel *betacoronavirus* severe acute respiratory syndrome coronavirus 2 (SARS-CoV-2) infection as the cause for the coronavirus disease 2019 (COVID-19) (1). During this time, despite global efforts for containment and the declaration of a pandemic on March 2020, there have been more than 126 million confirmed cases of COVID-19 worldwide, and over 3.5 million deaths reported to the World Health Organization (2).

Patients with SARS-CoV-2 infection can experience a range of clinical manifestations, from no symptoms to severe pneumonia, respiratory and/or multiple organ failure (1, 3, 4). Increasing evidence suggests that the immune response to SARS-CoV2 plays a critical role in the pathogenesis of COVID-19 disease. On one end of the spectrum, SARS-CoV-2 can disrupt normal immune responses, resulting in uncontrolled inflammation in severe and critical patients with COVID-19 (5). Specifically, lung infiltration and activation of pro-inflammatory myeloid cells such as monocytes, macrophages and neutrophils, is thought to play a key role in the cytokine storm syndrome and the hyper-inflammatory response observed in severe cases (6–8). On the other hand, adaptive immune responses elicited by emerging COVID-19 vaccines have shown to be highly protective against severe disease and mortality (9).

Understanding the immunopathology of SARS-CoV-2 can be harnessed for the identification of novel biomarkers for disease progression, as well as potential therapeutic targets for COVID-19. In this review, we summarize the characteristics of COVID-19 related dysregulation of the myeloid cell compartment, and discuss their potential use as biomarkers and future targets for therapeutic intervention.

## MDSC Definition and Functionality

Myeloid-derived suppressor cells (MDSCs) are defined as innate bone-marrow-derived immune cells suppressing effector T cell responses (10). MDSCs are a heterogeneous population mainly composed of two distinct subtypes, neutrophilic/granulocytic MDSCs (PMN-/G-MDSCs) and monocytic MDSCs (M-MDSCs) (10, 11). Differences compared to terminally differentiated granulocytes and monocytes respectively have been previously described in detail (11) but also key differences are summarized in **Table 1**. While initial investigations focused on T cells as targets of MDSC-mediated suppression, subsequent studies expanded this concept by showing that MDSCs are able to regulate a broad variety of adaptive (T cells, B cells) and innate (natural killer cells, macrophages, dendritic cells) immune cells (12, 13). Beyond dampening immune cell functionalities, MDSCs were further found to promote the development of regulatory T cells (14) and regulatory B cells (15). The effector mechanisms employed by MDSCs to control immune cell subsets depend on the MDSC subtype with PMN-MDSCs mainly use reactive oxygen species (ROS) and arginase I, whereas M-MDSCs use inducible nitric oxide synthase (iNOS) and arginase I to dampen bystander cells. In addition to these major suppressive mechanisms, other immuno-modulatory MDSC functions have been reported, including secretion of anti-inflammatory mediators such as interleukin-10 (IL-10), transforming growth factor beta (TGF-β) or Prostaglandin E2 (PGE2) or the tryptophan/kynurenine pathway through Indoleamine 2,3 dioxygenase (IDO) (12, 13, 16).

**Table 1:** Differences between MDSCs and other myeloid cells*.

Proposed differences between monocytes and M-MDSC	Suppressive function Reduced HLA-DR expression Increased iNOS expression Increased ARG1 expression Increased pSTAT3 expression Reduced IRF8 expression Increased cEBP/b expression Increased S100A8/9 expression Increased IL-4R (CD124) expression
Proposed differences between neutrophils/granulocytes and PMN-MDSC	Suppressive function Density (Ficoll gradients) Increased ROS expression Increased ARG1 expression Increased CD33 & CD66b expression

*non-exhaustive list.

**Figure 1:**
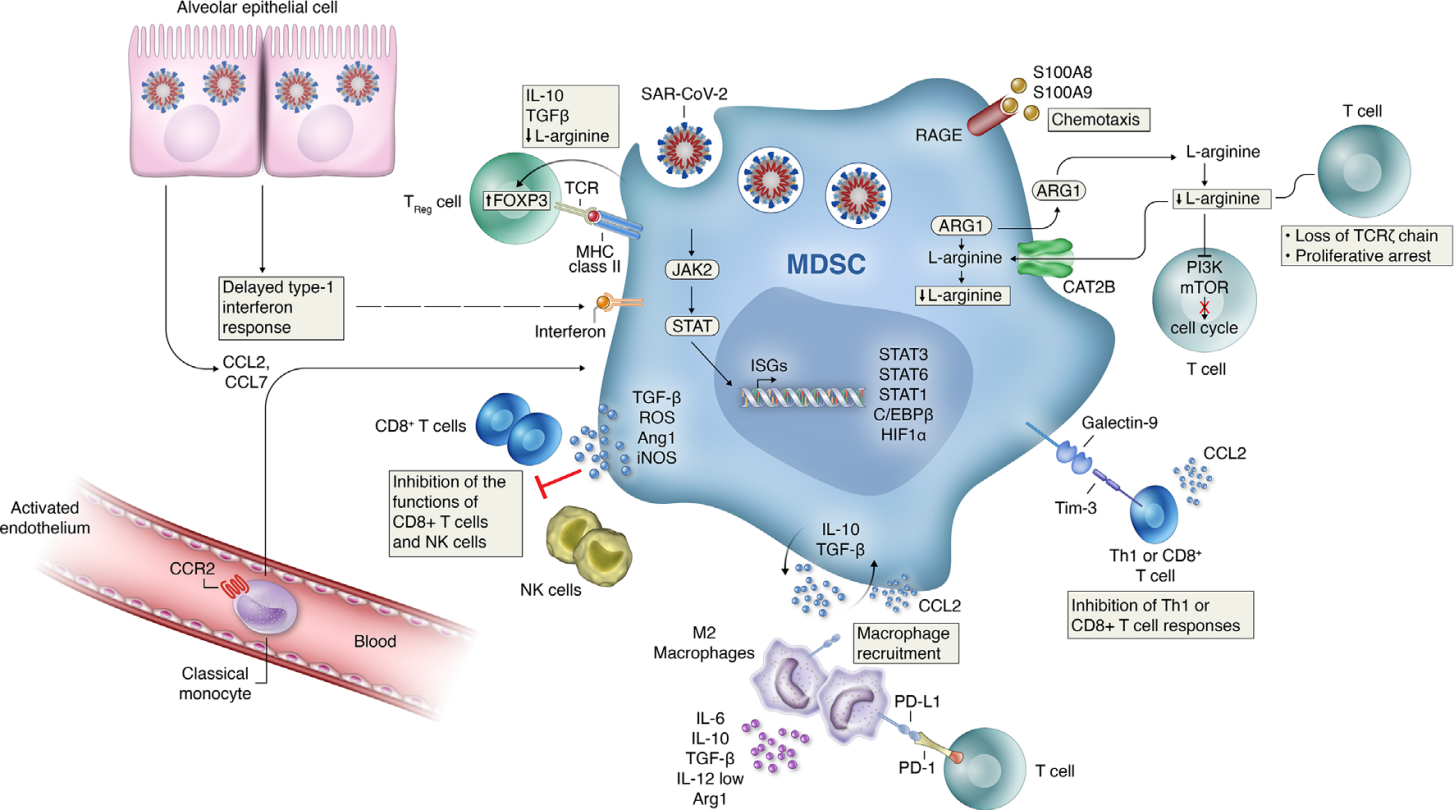
Mechanisms of MDSC-induced immune suppression and development of hyper-inflammation activation in COVID-19. Several mechanisms likely contribute to the MDSC-induced immune suppression and development of hyper-inflammation activation seen in patients with COVID-19. Delayed production of type I interferon leading to enhanced release of monocyte chemoattractants by alveolar epithelial cells leading to sustained recruitment of MDSCs into the lungs. TGFβ and IL-10 release by MDSCs can induce further inflammatory programs in resident (M2) macrophages while recruiting inflammatory monocytes, as well as granulocytes and lymphocytes from circulation. Signaling through activation of Janus kinase (JAK)–signal transducer and activator of transcription (STAT) pathways is necessary for MDSC expansion. Increased HIF1α expression can induce the transcription of inflammation related genes. The effector mechanisms employed by MDSCs to control immune cell subsets depend on the MDSC subtype with PMN-MDSCs mainly use reactive oxygen species (ROS) and arginase I, whereas M-MDSCs use inducible nitric oxide synthase (iNOS) and arginase I to dampen bystander cells. Increased PDL1 expression on recruited macrophages and MDSCs can directly decrease antigen-specific T-cell activation through interactions with the PD-1 receptor on T-cells. Increased signaling through the Galectin-9 and Tim-3 pathway can lead to the inhibition of Th1 or CD8+ T cell responses. Increased production of TGFb, ROS and L-arginine production by MDSC can inhibit the function of NK and CD8+ T cells during disease progression. Activated MDSCs contribute to the COVID-19 cytokine storm by releasing high amounts of pro- inflammatory cytokines.

While MDSCs were discovered in malignant diseases and the majority of studies assessed MDSCs in cancer conditions, emerging evidence shows that MDSCs are more diverse and are involved in inflammation, autoimmunity and infection (17). The factors inducing MDSC accumulation and suppressive function in these disease contexts remain only partially defined, but probably include a variety of microenvironmental factors including hypoxia, Granulocyte- and Granulocyte-Macrophage Colony Stimulating Factor (G-/GM-CSF), IL-6, Tumor necrosis factor alpha (TNF-α), Vascular Endothelial Growth Factor (VEGF), IL-1β and other cytokines, chemokines, damage-associated molecular patterns (DAMPs) and alarmins, such as High mobility group box protein 1 (HMGB1) or S100A8/9 (Calprotectin), checkpoint regulators, such as PD-1/PD-L1 and pathogen-associated molecular patterns (PAMPs), such as flagellin (12, 13, 16, 18, 19). At the transcriptional level, signaling through the transcription factors signal transducer and activator of transcription 3 (STAT3) and STAT5 are key for MDSC expansion (18) (**Figure 1**) whereas transcriptional regulators such as the Inhibitor of Differentiation 1 (Id1) have also been implicated in MDSC expansion (20).

MDSCs were studied in bacterial, viral, parasitic and fungal infections (21). Most so far studied infectious disease settings provided evidence for the accumulation of MDSCs in the peripheral blood and/or the affected tissue, yet the functional role of MDSCs has been reported as both detrimental by downregulating host defense or beneficial by dampening excessive infection-associated inflammation (or other less-defined mechanisms). In some infection models, the protective *vs.* harmful role of MDSCs is rather complex and depends on the animal species/model system used, the stage of disease, and the ratio/balance between pathogens, T cells and MDSC (21–23). Another layer of complexity in host-pathogen interactions is added by the fact that MDSCs as phagocytes can directly act anti-microbial or can be exploited by intracellular pathogens as survival niche. With regards to viral infections, most evidence for MDSC involvement exists for hepatitis B/C (HBV/HCV), human immunodeficiency virus-1 (HIV-1), herpes simplex virus (HSV) or influenza with indications that chronic, rather than acute viral infections induce MDSC expansion (24–26). Intriguingly, MDSC expansion in chronic HCV infection was shown to favor viral persistence (27), whereas in HBV infection MDSC were linked to a protective role by ameliorating hepatic tissue damage (28). In HIV infections, high numbers of MDSC were reported that correlated positively with viral loads, negatively with CD4^+^ T cell numbers and dropped upon antiviral therapy (29).

## Immunological Aspects of COVID-19

Despite a rapidly increasing number of publications on COVID immunopathogenesis, the precise mechanisms that govern the pathophysiology of the different disease courses of COVID-19 remain poorly defined owing to the complex multi-organ, co-morbidity-, age- and gender-dependent host and evolving viral nature of this condition. Peripheral blood immune signatures across COVID-19 patients revealed changes in both the innate and adaptive arm of immune responses, particularly in B and myelomonocytic cell composition, profoundly altered T cell phenotypes and selective cytokine/chemokine upregulation and SARS-CoV-2-specific antibodies (30). While most studies focused their attention on T cells, more comprehensive immune profiling approaches found that in severe cases the number of T and B lymphocytes, dendritic cells, natural killer (NK) cells, and HLA-DR^high^ expressing cells were found to be substantially decreased in COVID-19 disease (31). High-dimensional flow cytometry-analysis focusing on mononuclear phagocyte (MNP) lineages in SARS-CoV-2-infected patients with moderate and severe COVID-19 identified a redistribution of monocyte subsets toward intermediate monocytes and a general decrease in circulating DCs, which coincided with the appearance of MDSCs and a higher frequency of pre-DC2. Furthermore, the presence of a MNP profile was associated with a cluster of COVID-19 non-survivors (32).

Innate immune sensing serves as the first line of antiviral defense and is essential for immunity to viruses. Coronaviruses (CoVs) have evolved several mechanisms to inhibit IFN-I induction and signaling, e.g. suppression of MAV signaling (33, 34). Patients with severe COVID-19 demonstrate remarkably impaired IFN-I signatures as compared to mild or moderate cases and fail to elicit an early IFN-I response (35–37). Perhaps timing is key, as IFN is protective early in disease but later becomes pathogenic. Furthermore, while pathogenic CoVs block IFN signaling, they may actively promote other inflammatory pathways contributing to disease pathology. Among innate immune cells, particularly neutrophil counts were found to be significantly elevated in patients with COVID-19 and correlated with disease severity (38–40). An elevated neutrophil-to-lymphocyte ratio has been further suggested as clinical marker for predicting fatal complications related to Acute Respiratory Distress Syndrome (ARDS) in patients with COVID-19 (38). Increased production of pro-inflammatory cytokines and MDSCs inversely correlated with perforin-expressing NK and CD3+ T cells during disease progression (38, 39, 41). An early elevation in cytokine levels was associated with a maladapted immune response profile and worse disease outcomes (42). Elevated levels of cytokines, such as IP-10/CXCL10, interleukin-10 and interleukin-6, were shown to predict subsequent clinical progression (30).

There is increasing evidence to suggest that distinct innate immune responses, specifically, underlie the different clinical trajectories of COVID-19 patients and that the hyperinflammatory syndrome in severe COVID-19 results from a dysregulated host innate immune response (43). Transcriptomic, epigenomic, and proteomic analyses revealed widespread dysfunction of peripheral innate immunity in severe and fatal COVID-19, with the most profound disturbances including a prominent neutrophil hyper-activation signature and monocytes with anti-inflammatory features (7, 44, 45). Several studies have identified emergency myelopoiesis as a hallmark of severe or fatal COVID-19 (7, 45–47). Collectively, COVID-19 dysregulates both the innate and the adaptive immune system with a decrease of adaptive T cells and an increase of innate immune cell populations. However, the inter-connectedness of innate and adaptive immune changes remained largely elusive. Very recent studies provide evidence that MDSCs could represent that missing link as discussed below in detail.

## MDSCs in COVID-19

As outlined above, MDSCs are innate immune cells regulating (mostly downregulating) adaptive immune responses. MDSC activity can be enhanced by malignant or infectious triggers as described for a variety of viral, bacterial and fungal infections. Several recent reports have shown that patients with severe COVID-19 exhibit a dysregulated myeloid cell compartment, with increased MDSC levels and activity correlating with disease severity. In mild COVID-19 disease course, studies have reported an increase in HLA-DR^hi^CD11c^hi^ inflammatory monocytes with an interferon-stimulated gene signature, indicative of terminally differentiated monocytes, whereas severe COVID-19 was characterized by a lack of type I IFNs, high levels of HLA-DR^low^ classical monocytes and CD10^low^CD101^-^CXCR4^+/-^ neutrophils with an immunosuppressive profile in blood and lungs of severe cases, suggestive of emergency myelopoiesis (7, 47–50).

In severe COVID-19 patients with ARDS an increased ratio of MDSC to CD8 effector memory T cell was observed compared to patients hospitalized for COVID-19 moderate pneumonia, with COVID-19-related MDSC expansion directly correlating with lymphopenia and enhanced arginase activity (51). Marked expansion of MDSCs was observed, up to 90% of total circulating mononuclear cells in patients with severe disease, and up to 25% in the patients with mild disease with frequency decreasing with recovery (52). Granulocytic (neutrophil, eosinophil, and basophil) markers were enriched during COVID-19 and discriminated between patients with mild and severe disease, suggestive of PMN-MDSC activity (53). Increased counts of CD15^+^CD16^+^ neutrophils, decreased granulocytic expression of integrin CD11b, and Th2-related CRTH2 downregulation in eosinophils and basophils established a COVID-19 signature. Severity was also associated with emergence of PD-L1 checkpoint expression in basophils and eosinophils (53).

In summary, COVID-19 patients show a shift towards an immature myeloid cell profile in peripheral blood together with mature monocytes and segmented neutrophils, likely the result of emergency myelopoiesis in response to the significantly elevated levels of cytokines and other pro-inflammatory mediators in these patients. As myeloid cells are the main immune cell subsets associated with severe COVID-19, identification of their inflammatory and chemotactic gene signatures could be of potential prognostic as well as therapeutic relevance (54).

## MDSCs as Potential Biomarker in COVID-19

There is compelling evidence that primarily PMN-MDSC (55) but also M-MDSC subtypes accumulate in COVID-19 patients and are associated with distinct gene and protein signatures. Given the increase of circulating MDSCs in COVID-19 and a correlation with disease outcome, an obvious clinical implication is to consider their utilization as biomarkers of immune dysregulation in COVID-19.

PMN-MDSC expanded during COVID-19 in patients who required intensive care treatments, and correlated with IL-1beta, IL-6, IL-8, and TNF-alpha plasma levels (55, 56). The expression of lectin-type oxidized LDL receptor 1 (LOX-1) on PMN-MDSC, in particular, has been proposed to identify a subset of MDSCs with the most potent immunosuppressive properties, the elevation of which, was found to be more pronounced in patients with ARDS (57). Furthermore, a marked increase in Hexokinase II+ PMN-MDSC was found exclusively in the acute COVID-19 patients with moderate or severe disease (58). PMN-MDSC inhibited T-cell IFN-gamma production upon SARS-CoV-2 peptides stimulation, through TGF-beta- and iNOS-mediated mechanisms, possibly counter-acting virus elimination (55). An observed MDSC decline at convalescent phase was associated to a reduction in TGF-beta and to an increase of inflammatory cytokines in plasma samples (52). Finally, a multivariate regression analysis found a strong association between PMN-MDSC percentages and fatal disease outcome and PMN-MDSC frequencies were higher in non-survivors than survivors (55).

M-MDSCs were also found to expand in blood of COVID-19 patients, suppress T cells and strongly associate with disease severity. More specifically, a population of M-MDSC expressing high carnitine palmitoyltransferase I (CPT1a) and VDAC, were present in the PBMC of the acute COVID-19 patients and correlated with severity of disease (58). Furthermore, M-MDSC frequencies were elevated in blood but not in nasopharyngeal or endotracheal aspirates of COVID-19 patients compared to controls (59). M-MDSCs isolated from COVID-19 patients suppressed T cell proliferation and IFN-gamma production partly *via* an arginase-1 (Arg-1) dependent mechanism. Furthermore, these patients showed increased Arg-1 and IL-6 plasma levels. COVID-19 patients had fewer T cells, and displayed downregulated expression of the CD3zeta chain (59). In related studies, T cell proliferative capacity *in vitro* was significantly reduced among COVID-19 patients and could be restored through arginine supplementation (51).

Single-cell RNA sequencing (scRNAseq) data from bronchoalveolar lavage (BAL) also revealed the presence of neutrophils and macrophages as a hallmark of severe COVID-19 (54). Among the identified gene signatures, IFITM2, IFITM1, H3F3B, SAT1, and S100A8 gene signatures were highly associated with neutrophils, while CCL8, CCL3, CCL2, KLF6, and SPP1 were associated with macrophages in severe-COVID-19 patients. These findings are in agreement with high levels of calprotectin (S100A8/S100A9) found in plasma of severe cases (47). Genes associated with the inflammatory response and chemotaxis of myeloid cells, phagocytes, and granulocytes were among the top activated functional categories in BAL from severe COVID-19-affected patients (54). A lack of type I IFNs, reduced HLA-DR in myeloid cells and transient expression of IFN-stimulated genes characterized the transcriptome of patients with severe COVID-19 (50). Similarly, in a meta-analysis of transcriptomic data, the upregulation of the monocytic compartment in severe COVID-19, was dependent on the cytokines IL-6 and IL-10, and was characterized by broadly immunosuppressive properties and decreased responsiveness to stimulation (46). Myeloid cells of severe patients showed higher expression of pro-inflammatory cytokines and chemokines such as CXCL8 (60).

ScRNAseq profiling was further used to characterize the PBMC compartment of uninfected controls and COVID-19 patients and cells in paired broncho-alveolar lavage fluid (BALF) (61). A close association of decreased DCs and increased monocytes resembling MDSCs, correlated with lymphopenia and inflammation in the blood of severe COVID-19 patients. Those MDSC-like monocytes were characterized as ‘immune-paralyzed’. In contrast, monocyte-macrophages in BALFs of COVID-19 patients produced massive amounts of cytokines and chemokines, but secreted little interferons (61). In similar meta-analyses, an overall upregulation of immuno-inhibitory receptors mRNA during SARS-CoV-2 infection, expressed on both lymphoid and myeloid cells were upregulated in nasopharyngeal swabs and autopsies (e.g. BTLA, LAG3, FCGR2B, PDCD1, CEACAM1, CTLA4, CD72, and SIGLEC7), also directly correlated with viral levels (62). Integration of plasma proteomics with nine published scRNAseq datasets also revealed that disease severity in lung tissue is driven by myeloid cell phenotypes and cell-cell interactions with lung epithelial cells and T cells. Epithelial damage more specifically was associated with neutrophil infiltration (63).

Immature myeloid subsets and bronchoalveolar cells of critically-ill COVID-19 patients have been found to express HIF1alpha, a critical regulator of the differentiation and function of MDSCs, and transcriptional targets related to inflammation (CXCL8, CXCR1, CXCR2, and CXCR4); virus sensing, (TLRs); and metabolism (SLC2A3, PFKFB3, PGK1, GAPDH and SOD2) (64). The up-regulation and participation of HIF1alpha in events such as inflammation and immunometabolism make it a potential biomarker of COVID-19 severity. HIF1alpha and its transcriptionally regulated genes are also expressed in lung cells from severe COVID-19 patients, which may partially explain the hypoxia related events (64).

Calprotectin (S100A8/9) plasma level and a routine flow cytometry assay detecting decreased frequencies of non-classical monocytes have been shown to discriminate patients who develop a severe form of COVID-19, suggesting a prognostic value that deserves prospective evaluation (47). Elevated S100A-family alarmins in myeloid cells and marked enrichment of serum proteins that map to myeloid cells and pathways including cytokines, complement/coagulation, and fluid shear stress were also identified in pediatric MIS-C patients even in the absence of active infection (65). Soluble triggering receptor also expressed on myeloid cells had the best prognostic accuracy for 30-day intubation/mortality (66).

In summary, various observational retrospective investigations suggest that MDSCs, their subsets or MDSC-related markers and signatures (**Figure 1**) could serve as biomarkers for severe COVID-19 (Summarized in **Table 2**), yet prospective and multi-center biomarker-focused studies are required to (i) standardize MDSC assays used and (ii) define the prognostic and/or, if linked to therapeutic treatments, the predictive biomarker potential of MDSCs in COVID-19.

**Table 2:** Candidate myeloid biomarkers associated with COVID-19 disease severity.

Proposed Biomarker	Description of COVID-19 related findings	References
Neutrophil-to-lymphocyte ratio	Increased with severity	(38–40)
	Predicting ARDS complications	
MDSC to T-cell and NK-cell ratios	Increased with severity	(38, 39, 41, 51)
IP-10/CXCL10, interleukin-10 and interleukin-6	Increased with severity	(30)
	Prognostic markers	
HLA-DR^high^ expressing cells	Decreased with disease severity	(31)
IFN-I signature	Downregulated with severity	(35–37)
HLA-DR^low^ classical monocytes	Increased with severity	(7, 47–50)
CD10^low^CD101^-^CXCR4^+/-^ neutrophils	Increased with severity	(7, 47–50)
LOX-1 on PMN-MDSC	Increased with ARDS	(57)
Hexokinase II+ PMN-MDSC	Increased with severity	(58)
CD15^+^CD16^+^ CD11b^low^ neutrophils	Increased with severity	(53)
TGF-beta plasma levels	Increased with severity	(52)
		(55)
Arg-1 and IL-6 plasma levels	Increased with severity	(59)
M-MDSC expressing high CPT1a and VDAC	Increased with severity	(58)
HIF1-alpha expression	Upregulation with severity	(64)
Calprotectin (S100A8/9) plasma level	Prognostic marker for severe disease	(47)
Soluble triggering receptor	Prognostic marker for intubation/mortality	(66)

## MDSCs as Potential Therapeutic Target in COVID-19

Given the emerging role of MDSCs in COVID-19, another consequent question is how to therapeutically exploit and target this cell population. Based on insights from other, more established disease areas, such as oncology, therapeutic targeting of MDSCs can be achieved through different routes (16, 67): (i) drugs forcing MDSC differentiation into mature cells (e.g. vitamin D3 or retinoic acid), (ii) drugs inhibiting MDSC maturation from cellular precursors (e.g. bevacizumab, tyrosine kinase inhibitors, STAT3 inhibitors, MMP9 inhibitors), (iii) drugs reducing MDSC accumulation in peripheral organs (e.g. CXCR2/CXCR4 antagonists, 5-Flurouracil, Gemcitabine) or (iv) drugs affecting MDSC inhibitory functions (ROS scavengers, cyclooxygenase 2 (COX2) or phosphodiesterase type 5 (PDE5) inhibitors). Prostaglandin D2 (PGD2) has been proposed a key meditator of lymphopenia in COVID-19 and is known to upregulate M-MDSCs *via* the DP2 receptor signaling in group 2 innate lymphoid cells (ILC2). Targeting PGD2/DP2 signaling using a receptor antagonist such as ramatroban could be used in immunotherapy for immune dysfunction and lymphopenia in COVID-19 disease (68).

Alarmin S100A8 was robustly induced in SARS-CoV-2-infected animal models as well as in COVID-19 patients. Paquinimod, a specific inhibitor of S100A8/A9, could rescue the pneumonia with substantial reduction of viral loads in SARS-CoV-2-infected mice (69). A group of neutrophils that contributes to the uncontrolled pathological damage and onset of COVID-19 was induced by coronavirus infection. Paquinimod treatment could reduce these neutrophils and regain anti-viral responses, unveiling key roles of S100A8/A9 and aberrant neutrophils in the pathogenesis of COVID-19 and highlighting new opportunities for therapeutic intervention (69).

Therapeutic strategies targeting the migration/recruitment of myeloid cells from bone marrow as mentioned above could be considered for the treatment of COVID-19 induced hyper-inflammation and immune dysregulation (39). Inhibitors of CXCR2 or CCR2 and CCR5 may be able to reduce mobilization and migration of MDSC from the bone marrow to the circulation (70, 71). Following compassionate care treatment with the CCR5 blocking antibody leronlimab, a rapid reduction of plasma IL-6, restoration of the CD4/CD8 ratio, and a significant decrease in SARS-CoV-2 plasma viremia was observed. Consistent with reduction of plasma IL-6, single-cell RNA-sequencing also revealed declines in transcriptomic myeloid cell clusters expressing IL-6 and interferon-related genes (72).

Given the expression of inhibitory receptor upregulation observed in a variety of cell subsets during the progression of COVID-19 (62), targeting immuno-inhibitory receptors could also represent an effective therapeutic approach for the treatment of COVID-19 early and reversal of late immune dysregulation and suppression (73). Finally, reprogramming MDSCs by targeting immunometabolism and epigenetics may also holds promise in resolving lung inflammation associated with COVID-19 (74). As patients with severe COVID-19 have an increased inflammatory response that depletes arginine, and subsequently impairs T cell function, inhibition of arginase-1 and/or replenishment of arginine may be a potential future therapeutic approach in preventing/treating severe COVID-19 (75). Furthermore, the fatty acid transport protein 2 (FATP2), responsible for the uptake of arachidonic acid and for the subsequent synthesis of PGE2 was identified as a regulator of the suppressive functions of PMN-MDSCs (76). IDO dependent tryptophan metabolism is another pathway used by MDSCs to inhibit immune responses (77). Targeting metabolic mediators as FATP2 or enzymes such as IDO may be able to reverse MDSC induced suppression of virus-specific T-cell responses seen in severe COVID-19 cases.

Regarding potential targeting of MDSCs in COVID-19, it is essential to define the disease stage and disease severity level where such a therapeutic approach might have the greatest potential and would be beneficial rather than harmful to disease outcome. This consideration is key, as, in analogy to other viral infections (24–26). MDSCs may play a pathogenic or protective role depending on the time-course, pathogen load and severity of the individual disease condition. Given the COVID-19-associated lymphopenia, MDSCs were proposed as causal culprits to decrease T cells and thereby impair T cell-mediated host defense (**Figure 1**). On the other hand, MDSCs are capable of dampen overshooting tissue inflammation and might be beneficial at certain stages of disease. Therapeutic targeting would make sense at stages where MDSCs cause more harm than good and it is key to first identify those stages precisely.

## Conclusions

COVID-19 activates the innate immune system and suppresses adaptive T cell responses. MDSCs are key cellular players connecting innate and adaptive immunity. Both M-MDSCs and PMN-MDSCs accumulate in patients with COVID-19 and reflect disease outcome, but what does this mean for the future of COVID-19 diagnosis, monitoring and treatment? Currently, inflammation, cell-death- and coagulation-associated serum proteins such as CrP, LDH and IL-6 as well as D-Dimers are used to characterize COVID-19 severity and disease progression clinically; it remains to be assessed how MDSC frequencies in peripheral blood and/or airway fluids relate to these clinical serum markers and whether combined/composite biomarker scores composed of both serum proteins and cells (PMN-MDSCs and/or M-MDSCs) could be superior than clinical serum markers alone to monitor and predict the outcome and treatment response in COVID-19. Targeting MDSCs as future therapeutic approach in COVID-19 is farer away, yet could add substantial value, particularly in combination with other immunomodulatory drugs, such as cytokine blockers.

## Author Contributions

MR contributed to manuscript research and writing. FS contributed to manuscript research and writing. DH contributed to manuscript research and writing. All authors contributed to the article and approved the submitted version.

## Conflict of Interest

Authors MR, FS and DH were employed by the company Novartis.
